# Consequences of delay in screening, monitoring, and treatment of angiomyolipoma and tuberous sclerosis: A case report 

**DOI:** 10.5414/CN109382

**Published:** 2018-04-27

**Authors:** Tanjala T. Gipson

**Affiliations:** TSC Center of Excellence, LeBonheur Children’s Hospital, Boling Center for Developmental Disabilities, Memphis, TN, USA

**Keywords:** angiomyolipomas, everolimus, multidisciplinary treatment, tuberous sclerosis complex

## Abstract

Background: Tuberous sclerosis complex (TSC) is a multisystem disorder that results in tumor growth in various organs. TSC can affect the kidneys in the form of renal angiomyolipomas and cysts that can lead to chronic kidney disease. Case presentation: A 38-year-old woman was referred to Kennedy Krieger Institute for comprehensive TSC management. Before referral, the patient had gone most of her life without a definite diagnosis of TSC despite visually-prominent signs such as forehead plaques, facial angiofibromas, and ungual fibromas. Eventually, complications of the disease led to the patient requiring hemodialysis at age 34 and a complete bilateral nephrectomy at age 36. However, the patient was not diagnosed with TSC until an evaluation at the National Institutes of Health at age 37. After becoming a patient at our clinic, a multidisciplinary approach was taken to provide comprehensive care by including various disciplines such as nephrology, neurology, pulmonology, ophthalmology, dentistry, dermatology, and cardiology. Discussion and conclusion: TSC consensus recommendations aid in diagnosis, monitoring, and treatment of TSC and its associated manifestations, including those involving the kidneys. Our case underscores the importance of early identification of TSC to prevent future complications and promotes use of a multidisciplinary team to provide comprehensive care.

## Background 

Tuberous sclerosis complex (TSC) is an autosomal dominant genetic disorder affecting ~ 1 in 6,000 births [1, 2]. It is caused by mutations in *TSC1* or *TSC2* genes, which result in overactivation of the mammalian target of rapamycin (mTOR) pathway and subsequent tumor growth in multiple organs [3]. Angiomyolipomas develop in the kidneys in up to 80% of patients [4]. In the brain, cortical dysplasias appear in most patients and are associated with epileptogenesis and developmental disorders [5, 6]. Subependymal giant cell astrocytomas, which develop from subependymal nodules, occur in up to 20% of patients [7]. Tumors can also develop in other organs and include lymphangioleiomyomatosis (LAM) in the lungs, various skin lesions, retinal hamartomas, and cardiac rhabdomyomas [8]. 

Consensus recommendations published in 2012 provide guidance on the diagnosis and management of TSC [8, 9]. A multidisciplinary approach is recommended in disease surveillance and management [9]. Early diagnosis and treatment of TSC is paramount, as delays in treatment can lead to the development of complications. In particular, renal angiomyolipomas can encroach on renal tissue, leading to end-stage renal disease [4]. Larger renal angiomyolipomas lead to a higher risk of aneurysm and subsequent hemorrhage [4]. As a result, they are the most common cause of TSC-related mortality in adults [10]. 

The case presented herein demonstrates the long-term consequences for a patient whose TSC had not been diagnosed until later in life and the multidisciplinary approach taken to manage her treatment. This case is valuable to nephrologists to emphasize that drastic renal complications can result from such a delay in diagnosis – complications that may have been otherwise preventable or manageable with an earlier diagnosis of TSC. 

## Case presentation 

A 38-year-old woman was referred to Kennedy Krieger Institute a year after being officially diagnosed with TSC at the National Institutes of Health (NIH) in October 2012. Before her diagnosis, the patient had several TSC-related manifestations that should have solidified diagnosis much earlier in her life. She had TSC-associated skin lesions, such as forehead plaques that were apparent as an infant and facial angiofibromas that developed later in her childhood. In her teenage years, she developed periungual fibromas on her foot that would only be removed by a podiatrist in 2003 when she was an adult. 

In October 2009, at age 34, the patient had been put on hemodialysis; however, incomplete records received by our institute did not identify the indication for this. At least 1 large (3.8-cm) renal angiomyolipoma in the left kidney and multiple bilateral cysts were identified in 2011. These disease manifestations led the patient to undergo a bilateral nephrectomy later that year at age 36, and renal transplantation was considered as an option in the following year. She had also developed cardiac issues such as hypokinesis and low ejection fraction (40%), likely the result of renal complications. In addition to renal and cardiac issues, lung deterioration was observed as early as 2011. In October 2011, the patient presented with interstitial and alveolar infiltrates with bilateral pleural effusion. Diffuse lung cysts were observed in September 2012. 

In October 2012, she was evaluated and diagnosed with TSC at the NIH. Her diagnosis was based on multiple manifestations of TSC identified during her evaluation. Numerous types of TSC-associated skin lesions were identified, including facial angiofibromas, cephalic plaques, oral fibromas, shagreen patches, ungual fibromas, and confetti skin lesions. Dental pitting was also observed. Brain magnetic resonance imaging (MRI) revealed evidence of bilateral abnormal gray/white matter and bilateral subependymal nodules. Two retinal hamartomas were found in her left eye. 

The patient’s lung function subsequently declined, and in 2013, while being treated at Johns Hopkins Hospital, she was indicated for lung transplantation. However, there was a decision to wait for further decline because of the possibility of recurrence. She was also switched from hemodialysis to peritoneal dialysis to lessen fatigue. 

The patient’s first visit to our clinic occurred in September 2013, at which point she underwent an initial evaluation for comprehensive management of TSC. After examination and reviewing her past medical history, we found that there were no concerns regarding epilepsy or neurodevelopmental delay. Upon lung examination, decreased breath sounds were noted, and it was recommended to start everolimus 5 mg daily to slow the progression of LAM and potentially improve lung function; however, she elected not to initiate treatment at that time. She was listed for renal transplantation pending results of pulmonary testing. A full dental consultation with follow-ups every 6 months was also recommended, in particular for sealant of dental pits. 

In a subsequent visit in February 2014, abdominal imaging was recommended to monitor a previously-detected liver angiomyolipoma. After pulmonary tests, follow-up with other disciplines was recommended, including nephrology, neurology, pulmonology, ophthalmology, dentistry, dermatology, and cardiology. At this time, everolimus was recommended at 10 mg daily for treating pulmonary LAM, and the patient agreed to initiate treatment after this visit. 

After 1 day of taking everolimus 10 mg, the patient experienced dizziness and hypotension and self-discontinued treatment. Upon her visit in July 2014, it was decided to restart everolimus at 5 mg once daily, and midodrine was initiated for hypotension. The patient did not experience dizziness at the 5-mg dose. 

The patient’s next visit at our clinic would not be until March 2015. However, before this visit, she was scheduled for renal transplantation surgery that was stopped because of difficulties with anesthesia. As a result of depressed mood after her canceled renal transplantation, she self-discontinued everolimus again. As a result, pulmonary nodules and cysts throughout the lungs were observed with computed tomography, accompanied with worsening pulmonary function, and were likely attributable to the interruption of everolimus. We recommended she restart everolimus 5 mg once daily, and at her next visit focus on counseling and medication for depressed mood. As of July 2015, her mood has improved, and she is continuing everolimus 5 mg, with amenorrhea reported as the only noticed side effect. 

## Discussion 

The case study presented here demonstrates the importance of diagnosing and monitoring TSC as early as possible. Previous work has identified patients who go undiagnosed for years despite having visible signs of TSC early in life, but it does not go into extensive detail regarding the consequences of delayed diagnosis and management in these patients [11]. An earlier diagnosis might have prevented some of the complications experienced by our patient. The 2012 TSC consensus recommendations provide clinical criteria for diagnosis, which include major and minor features (Table 1) [8]. Skin lesions are one of the most visible early signs of TSC [12]. From infancy to adolescence, our patient exhibited forehead plaques, angiofibromas, and ungual fibromas. As major features according to consensus recommendations, these 3 manifestations would have solidified a definite diagnosis of TSC much earlier in her life and prompted screening for other TSC-associated manifestations, particularly those affecting renal function. While the patient’s complete history was unavailable to us, it is likely that her dialysis treatment was needed as a direct result of renal angiomyolipomas and cysts encroaching on renal tissue, leading to chronic kidney disease [13]. Earlier management of these manifestations might have prevented the need for complete bilateral nephrectomy. 

Consensus recommendations provide guidance on how to monitor and manage TSC-associated renal manifestations. For example, after definitive diagnosis, renal function should be assessed annually, and abdominal MRI should be conducted every 1 to 3 years to assess renal lesions (Table 2) [9, 13]. These recommendations also advocate the use of mTOR inhibitors as first-line treatment in asymptomatic cases of growing angiomyolipomas (> 3 cm in diameter) and selective embolization or kidney-sparing resection as second-line therapy. Embolization (followed by corticosteroids for 7 days to mitigate postembolization syndrome) [14] is recommended as first-line therapy in cases of acute renal hemorrhage. In general, nephrectomy should be avoided [9, 13]. 

It was important for this patient to receive a multidisciplinary approach to treatment to evaluate other, nonrenal TSC-related manifestations. At our clinic, we ensured follow-up from multiple disciplines. This approach encouraged greater monitoring in areas such as lung function, as lung deterioration due to LAM led to an indication for lung transplantation. Our approach to this deterioration was to use an mTOR inhibitor to potentially slow this decline. mTOR inhibitors have previously been shown to improve or stabilize lung function in patients with LAM and are also recommended by the 2012 TSC Consensus Conference for this use [9, 15, 16]. Multidisciplinary care of patients with TSC also requires adequate coordination among specialties to ensure that caregivers of the various disciplines are seeing the entire picture and do not lose track of the patient. This can be achieved if care is coordinated from a single service, such as a specialized TSC clinic. 

It is also important to emphasize compliance with treatment. On several occasions, our patient discontinued everolimus voluntarily because of adverse events. After one of these instances, lung function further deteriorated. Adverse events common with mTOR inhibitors include stomatitis, upper respiratory tract infections, and increased blood cholesterol [17]. Adverse events should be managed with careful monitoring and use of dose reductions or interruptions where necessary. 

In conclusion, we feel that this case outlines the importance of early and accurate diagnosis of TSC and the possible complications, particularly kidney-related, that can result when diagnosis is delayed. Our case also emphasizes the importance of a multidisciplinary approach to treating TSC, since such a multisystemic disease requires input and monitoring from a variety of disciplines in order to ensure adequate care. 

## Acknowledgment 

Medical writing and editorial assistance was provided by Traci Stuve, MA, and Robert Schoen, PharmD, of ApotheCom (Yardley, PA, USA). 

## Funding 

Medical writing assistance was supported by Novartis Pharmaceuticals Corporation. 

## Conflict of interest 

Dr. Gipson served as a principal site investigator for a Novartis study (EXIST-3); funding was provided to her institution only. 

**Table 1.** Clinical diagnostic criteria for tuberous sclerosis complex: 2012 consensus guidelines [8].

Major features	Minor features
Hypomelanotic macules (≥ 3, at least 5 mm in diameter)	“Confetti” skin lesions
Angiofibromas (≥ 3) or fibrous cephalic plaque	Dental enamel pits (> 3)
Ungual fibromas (≥ 2)	Intraoral fibromas (≥ 2)
Shagreen patch	Retinal achromic patch
Multiple retinal hamartomas	Multiple renal cysts
Cortical dysplasias	Nonrenal hamartomas
Subependymal nodules	
Subependymal giant cell astrocytoma	
Cardiac rhabdomyoma	
Lymphangioleiomyomatosis	
Angiomyolipomas (≥ 2)	

Definite diagnosis: Two major features or 1 major feature with ≥ 2 minor features. Possible diagnosis: Either 1 major feature or ≥ 2 minor features.

**Table 2.** Kidney-related surveillance and management recommendations [9, 13].

Newly diagnosed or suspected	TSC diagnosed with definite or possible TSC
Surveillance of kidneys	
Obtain MRI of the abdomen to assess for the presence of angiomyolipoma and renal cysts. Screen for hypertension by obtaining accurate blood pressure. Evaluate renal function by determining GFR.	Obtain MRI of the abdomen to assess angiomyolipoma progression and renal cystic disease (every 1 to 3 years, life-long). Assess renal function (GFR and blood pressure) at least annually.
Clinical presentation	Recommendation
Management recommendations for renal angiomyolipoma	
Angiomyolipoma with acute hemorrhage	Embolization (followed by corticosteroids for 7 days to mitigate postembolization syndrome) [14]. Embolization should be as selective as technically feasible to preserve renal parenchyma. Avoid nephrectomy.
Asymptomatic, growing angiomyolipoma > 3 cm in diameter.	First-line: mTOR inhibitor. Second-line: selective embolization or kidney-sparing resection.

GFR = glomerular filtration rate; MRI = magnetic resonance imaging; mTOR = mammalian target of rapamycin; TSC = tuberous sclerosis complex.
